# Diaphragmatic lipoma; A benign condition necessitating an aggressive management

**DOI:** 10.1016/j.ijscr.2019.10.031

**Published:** 2019-10-23

**Authors:** Aram Baram, Fahmi Hussein Kakamad, Kizhan Abdalla Hama Salih, Dezhin Faeq Rashid, Rawand Abdulrahman Essa

**Affiliations:** a College of Medicine, Department of Cardiothoracic and Vascular Surgery, University of Sulaimani, Kurdistan Region, Iraq; b Kscien Organization For Scientific Research, Hamdi Street, Sulaimani, Kurdistan Region, Iraq; c Shar Teaching Hospital, Department of Cardiothoracic and Vascular Surgery, Sulaimani, Kurdistan Region, Iraq

**Keywords:** Diaphragm, Lipoma, Malignancy, Hernia

## Abstract

**Introduction:**

Primary diaphragmatic lipoma (PDL) is a very rare condition. The aim of this paper is to report a case of diaphragmatic lipoma presenting as a case of diaphratmatic hernia with a brief literature review. A 73-year-old female presented with chronic dry cough for three month duration. The patient was stable, there was no significant finding on general, chest and abdominal examination. Chest-x-ray showed vague shadow above the left dome of the diaphragm. Computed tomography scan (CT scan) of the chest revealed a round mass with features consistent with either diaphragmatic hernia or malignancy. The patient underwent resection under general anesthesia and histopathological examination confirmed the diagnosis of PDL.

**Conclusion:**

Although PDL is a benign condition, in most of the time, it needs aggressive management because of the possibility of malignancy or other critical diseases.

## Highlights

• Primary diaphragmatic lipoma is a very rare condition.
• It may present with logically unrelated signs and symptoms.
• The aim of this paper is to report a case of diaphragmatic lipoma presenting as a case of diaphratmatic hernia.

## 1 Introduction

Diaphragm, the muscular barrier separating the chest from the abdomen, is considered as one of the most unaccustomed sites for primary tumor occurrence regardless of the nature of the tumor. Among those odd occasions, the primary benign tumors most commonly detected are bronchogenic and mesothelial cysts, while metastatic benign tumors include endometriosis [1,2]. They yield surgical intervention if they cause symptoms or whenever concern for malignant transformation is raised. The malignant tumors of the diaphragm include primary, metastatic, or direct extension of the tumor from adjacent structures, common examples being rhabdomyosarcom and myosarcoma [1].

Primary diaphragmatic lipomas are usually auxiliary findings. The first case of PDL was found in 1886 by Francis W. Clark in the postmortem examination of a 65-year-old female who died from the complication of intracapsular fracture of femur head [3]. Lipomas are soft masses of adipose (fat) cells with a capsule made of a thin layer of fibrous tissue. They are most commonly found in the subcutaneous tissue of the head, neck, and shoulders. The masses are often benign and develop between the fifth to the seventh decades of life [4].

The aim of this study is to report and discuss a case of diaphragmatic lipoma in line with SCARE guideline with a brief literature review [5].

## 2 Patient information

A 73-year-old housewife female presented with chronic dry cough for three month duration associated with episodes of excessive salivation. She reported attacks of shortness of breath and cyanosis especially during sleep. She was a known case of controlled essential hypertension and she had history of a chest wall lesion before one year which was resected and later on she was diagnosed as case of basal cell carcinoma. She also had history of open cholecystectomy, caesarian section and laminectomy.

## 3 Clinical findings

The patient was conscious, stable, there was no significant finding on general, chest and abdominal examination. Blood pressure: 160/80 mmHg, pulse rate 80 beats per minutes, respiratory rate 15 cycles per minute, temperature 37.3C.

## 4 Diagnostic assessment

**Fig. 1:**
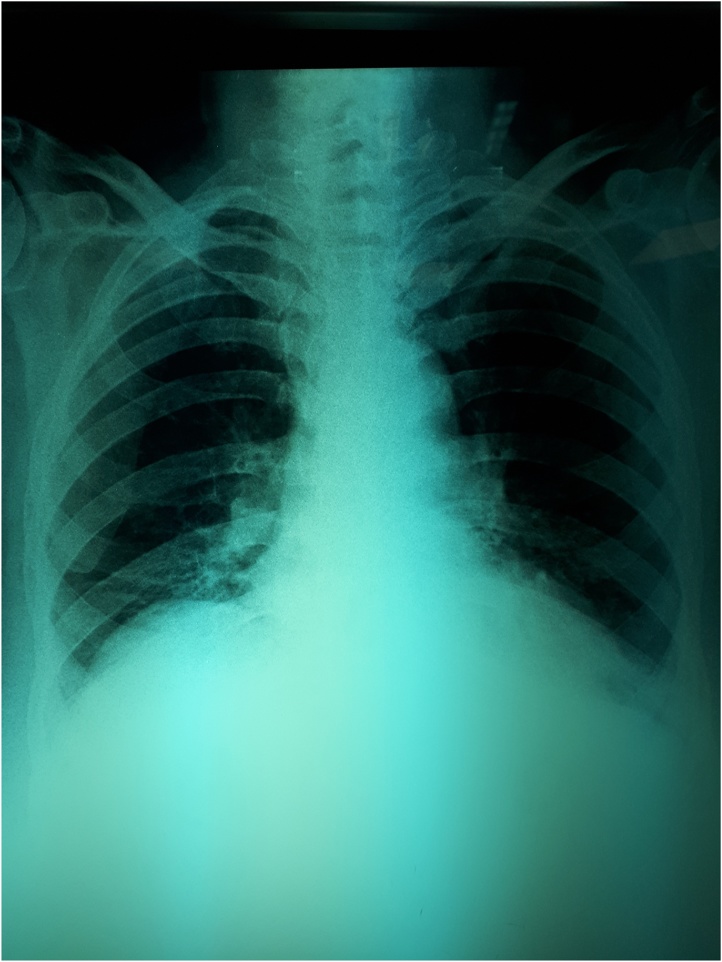
Plain posteroanterior chest radiograph showing a vague mass behind left cardiac shadow.

**Fig. 2:**
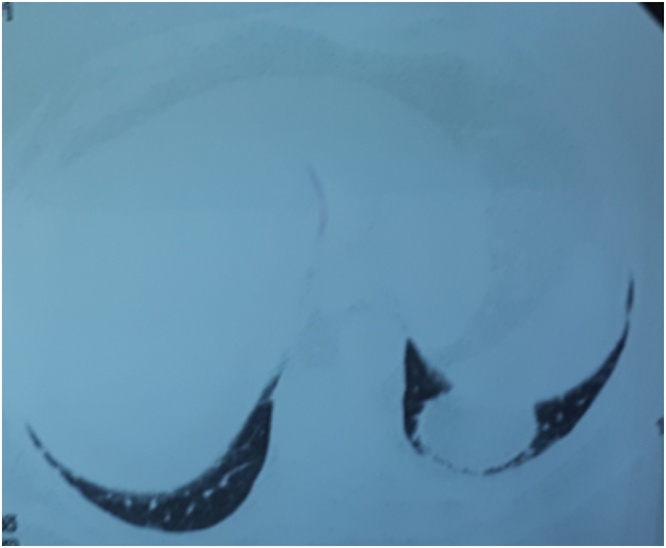
Computed tomography scan of the chest showing round mass with fat attenuation attached to the left dome of the diaphragm.

complete blood counts, renal function tests, serum electrolyte, electrocardiography were normal. Chest-x-ray showed vague shadow above the left dome of the diaphragm (Fig. 1). Bronchoscopy was also normal. Echocardiography showed hypertensive heart disease with impaired left ventricular diastolic function, ejection fraction 65%. Computed tomography scan of the chest revealed a round mass which was in continuity with the diaphragm with fat consistency, features consistent with either diaphragmatic hernia with omentum herniation into the chest cavity or malignancy (Fig. 2).

## 5 Therapeutic intervention

**Fig. 3:**
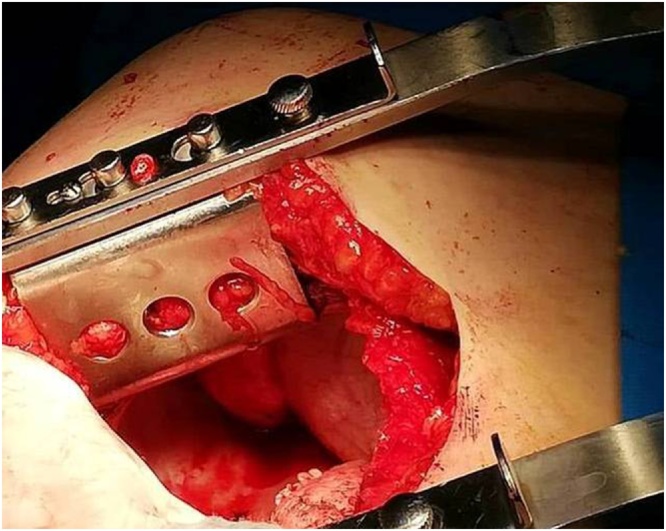
Intraoperative picture of the pathology.

**Fig. 4:**
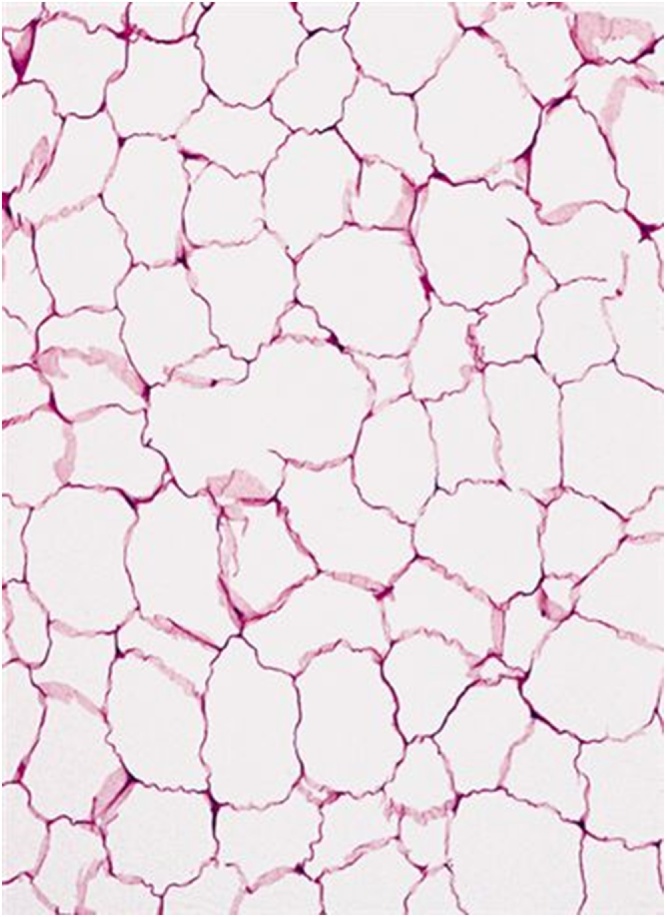
Cluster of mature adipocytes separated by fibrous tissues.

The patient was prepared for general anesthesia. Under general anesthesia, in right lateral position, left side classical posterolateral incision was done. A soft mass was found attached to the diaphragm (Fig. 3). It was totally resected, thoracostomy tube was left. The wound was closed in the classical way. The histopathological examination of the specimen showed features consistent with PDL (Fig. 4).

## 6 Follow-up and outcomes

The patient was healthy two months after the operation. All the symptoms were subsided.

## 7 Discussion

Primary diaphragmatic tumor is an extremely rare tumor, being benign is even rarer. PDLs are ovoid, encapsulated, soft tumors, mostly occur in middle age obese patients with equal gender distribution [6]. However, PDL has been reported in different age groups ranging from seven months to 85 years [1,4]. Cheon et al found a PDL in a 4-year-old female child during diagnostic work up for dry cough [7]. The current case was an old age female with normal body weight. The macroscopic features were consistent with lipoma. PDLs prevailingly affect the left side (ratio, 2 to 1) and posterolateral surface of the diaphragm (as in the present case), rarely they occur bilaterally [1,6]. In the series of Castillo and Shirkhoda, among 5 cases, one of them (20%) had bilateral PDL [2].

William and Parson have classifies PDLs based on their anatomical location into: (1) Intrathoracic lipoma: situated completely within the thoracic cavity, attaching to the diaphragm in a sessile or pedunculated form, developing from subpleural mature fatty tissues. (2) Hourglass thoracic lipoma: those possessing an intrathoracic and an extrathoraic portion, developing from embryonally undifferentiated tissue, (a) cervicomediastinal: going from the mediastinum to the neck. (b) transmural: going through the chest wall, commonly an intercostal space, seldom through the sternum [4,8,9]. The current case was intrathorscic sessile lipoma without extension outside the thoracic cavity.

Clinically, they may stay dormant with occasional symptomatic patients complaining of cough, dyspnea, chest pain, back and shoulder pain, hemoptysis, cardiac problems, obstructive sleep apnea, and even becoming fatal in case of diaphragmatic rupture [6,7,10]. Aydin and associates reported five symptomatic patients of PDL. The most common symptom was chest pain which was reported in three cases (60%), followed by back pain, cough and hemoptysis, each one was found in two cases (20%) [6]. Papachristos and his colleagues reported a 66-year-old female presented with pleuritic chest pain, shortness of breath, wheeze, strider, and respiratory failure [10]. On the other hand, asymptomatic PDLs were more frequently reported in the literature even when the mass enlarged enough to occupy half of the affected hemithorax [3,4]. Margiotta and colleagues published the case summery of a victim of road traffic accident who burned to death. She was an 85-year-old dead on arrival female, on autopsy examination, there was a big mass (15 × 15 × 20 cm) occupying half of the right hemithorax. The family denied any symptom related to the cardiovascular and respiratory systems [4]. The current patient complained from shortness of breath, cough, occasional excessive salivation and features of obstructive sleep apnea. The symptoms completely subsided after the operation.

Noninvasive imaging modalities such as chest-x-ray, ultrasound, CT scan or magnetic resonance imaging (MRI) are considered as gold standard for the diagnosis [2]. PDLs are often misapprehended for diaphragmatic hernias, liposarcoma, localized eventrations, or local elevation of the diaphragm by intra-abdominal pathologies. Differentiating these entities presents a great difficulty in diaphragmatic imaging [1,9]. Papachristos and associates labeled the chest-X-ray of the their patient as a normal image, retrospectively they found that there was elevated left hemi-diaphragm, unfolded thoracic aorta and widening mediastinum [10]. In the report of Cheon and colleagues, chest-X-ray, CT scan of the chest and upper abdomen showed a mass on the liver and they opened the patient through right subcostal incision (abdominal approach) and they were surprised by a large PDL [7]. The current case was provisionally diagnosed as a case of diaphragmatic hernia with omental herniation into the chest, although malignancy was not excluded.

Surgical intervention is advocated for all cases as the probability of early stage liposarcoma cannot be eliminated [7]. Open classical posterolateral incision is the most prevalent approaches practiced [10]. Among five cases, Aydin and colleagues managed one case (20%) with video assisted thoracoscopic surgery with the same outcome [6]. This patient was managed by posterolateral incision. Outcome is excellent, neither morbid complication nor recurrence has never been reported in the literature.

In conclusion, PDL is a very rare condition. Although it is a benign condition, in most of the time, it needs aggressive management because of the possibility of malignancy or other critical diseases.

## Sources of funding

No source to be stated.

## Ethical approval

Approval has been taken from Kscien centre.

## Consent

Consent has been taken from the patient and the family of the patient.

## Author contribution

Aram Baram: Surgeon performed the operation and follow up.

Fahmi H. Kakamad: Writing the manuscript and follow up.

Kizhan abdalla hama salih: Writing the manuscript, follow up.

Rawand Abdulrahman Essa: Revising the manuscript, follow up of the patient.

Dezhin Faeq Rashid: Writing the manuscript, follow up of the patient.

## Registration of research studies

Not applicable.

## Guarantor

Fahmi Hussein kakamad.

## Provenance and peer review

Not commissioned, externally peer-reviewed.

## Declaration of Competing Interest

There is no conflict to be declared.
